# Predictive Value of Two-Dimensional Speckle-Tracking Echocardiography in Patients Undergoing Surgical Ventricular Restoration

**DOI:** 10.3389/fcvm.2022.824467

**Published:** 2022-03-21

**Authors:** Olena Nemchyna, Natalia Solowjowa, Michael Dandel, Yuriy Hrytsyna, Julia Stein, Jan Knierim, Felix Schoenrath, Felix Hennig, Volkmar Falk, Christoph Knosalla

**Affiliations:** ^1^Department of Cardiothoracic and Vascular Surgery, German Heart Center Berlin, Berlin, Germany; ^2^Cardio Centrum Berlin, Berlin, Germany; ^3^DZHK (German Centre for Cardiovascular Research), partner site Berlin, Berlin, Germany; ^4^Department of Cardiothoracic Surgery, Charité - Universitätsmedizin Berlin, corporate member of Freie Universität Berlin, Humboldt-Universität zu Berlin, and Berlin Institute of Health, Berlin, Germany; ^5^ETH Zurich, Department of Health Sciences and Technology, Translational Cardiovascular Technology, Zurich, Switzerland; ^6^Charité - Universitätsmedizin Berlin, corporate member of Freie Universität Berlin, Humboldt-Universität zu Berlin, and Berlin Institute of Health, Berlin, Germany

**Keywords:** surgical ventricular repair, surgical ventricular restoration, heart failure, left ventricular aneurysm, speckle-tracking echocardiography (STE), outcome study

## Abstract

**Objectives:**

Parameters of left ventricular (LV) mechanics, obtained from speckle-tracking echocardiography (STE), were found to be of prognostic value in patients with heart failure and those who underwent cardiac surgery. This study aimed to assess the value of STE in patients scheduled to undergo surgical ventricular restoration (SVR).

**Methods:**

A total of 158 consecutive patients with baseline STE who underwent SVR due to an LV anteroapical aneurysm were included in the analysis. Preoperative longitudinal STE parameters were evaluated for their association with an outcome, defined as all-cause mortality, LV assist device implantation, or heart transplantation. The echocardiographic follow-up to assess the change in the regional function of the segments remote from the aneurysm was performed in 43 patients at a median of 10 months [interquartile range (IQR): 6–12.7 months] after SVR.

**Results:**

During a median follow-up of 5.1 years (IQR: 1.6–8.7 years), events occurred in 68 patients (48%). Less impaired mean basal end-systolic longitudinal strain (BLS) with a cutoff value ≤ −10.1 % demonstrated a strong association with event-free survival, also in patients with an LV shape corresponding to an intermediate shape between aneurysmal and globally akinetic. Initially hypo- or akinetic basal segments with preoperative end-systolic strain ≤ −7.8% showed a greater improvement in wall motion at the short-term follow up.

**Conclusion:**

Patients with less impaired preoperative BLS exhibited a better event-free survival after SVR, also those with severe LV remodeling. The preserved preoperative segmental longitudinal strain was associated with a greater improvement in regional wall motion after SVR. BLS assessment may play a predictive role in patients with an LV anteroapical aneurysm who are scheduled to undergo SVR.

## Central Message

The preserved longitudinal strain of basal LV segments is associated with an improvement in regional LV function after surgical ventricular restoration and with better event-free survival.

## Perspective Statement

Preoperative assessment of basal longitudinal strain can be used to predict the improvement in regional LV function and event-free survival of patients undergoing surgical ventricular restoration. Performing quantitative analysis of LV mechanics using two-dimensional speckle-tracking may become an important component of the integrative approach in SVR decision-making in these patients.

## Introduction

Left ventricular (LV) remodeling after myocardial infarction leads to enlargement of the left ventricle, a change in its geometry, and an increase in wall tension, which continuously impairs the LV systolic function and clinically results in progression of heart failure (HF) (1). Surgical ventricular restoration (SVR) aims to reduce LV volume, normalize LV geometry, enhance LV performance, and prevent the progression of remodeling. The removal of extensive scar tissue and an aneurysmal sac reduces wall stress, improves the ejection fraction (EF), and increases the stroke work index (2). Despite being a convincing pathophysiological concept, the benefits of SVR in improving survival are less recognized. In the multicenter randomized Surgical Treatment for Ischemic Heart Failure trial (STICH), coronary artery bypass surgery (CABG) with concomitant SVR in patients with ischemic cardiomyopathy did not show better outcomes than CABG alone (3). Furthermore, no prognostic benefit was demonstrated as regards the presence of viable myocardium (4) or the visual assessment of regional LV function (5). In a subsequent subanalysis, a less dilated left ventricle, a less impaired EF, and an adequate volume reduction were identified as predictors of favorable outcomes after SVR (6). In nonrandomized studies, we and others demonstrated a benefit of SVR and found additional predictors of adverse outcomes (7–12). Currently, there are no clear guidelines for the decision-making process in patients with an LV aneurysm. Therefore, additional criteria for better procedural planning, patient selection, and prediction of outcome after SVR are required.

Two-dimensional speckle-tracking echocardiography (STE) is a useful technique for evaluating the regional and global LV function in patients with coronary artery disease. Global longitudinal strain (GLS) was found to play a predictive role in patients with HF (13) and in those scheduled for cardiovascular surgery (14). Longitudinal and radial LV strain can be used to detect viable myocardium in patients with chronic ischemic systolic dysfunction and is comparable to cardiac MRI (15).

The aim of this study was to evaluate the value of preoperative longitudinal STE parameters in patients who underwent SVR due to an anteroapical LV aneurysm. We hypothesized that the regional function at the basal level of the left ventricle is a valuable factor in reverse remodeling after SVR and, therefore, may be of prognostic value for these patients.

## Methods

### Study Population and Outcome

**Figure 1:**
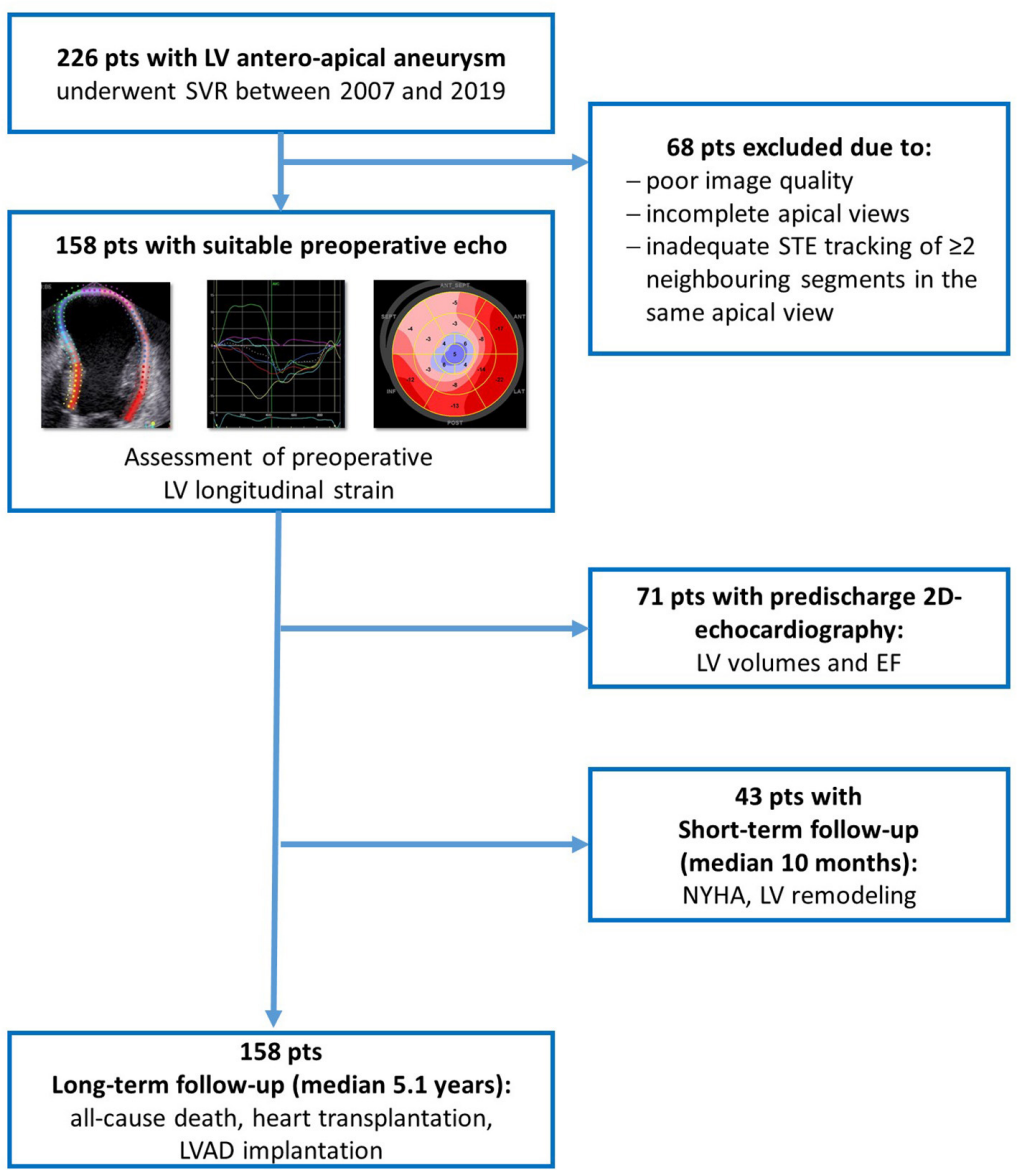
Study flowchart.

A total of 226 patients with a postischemic anteroapical LV aneurysm underwent SVR at our center between November 2007 and January 2019. In total, 158 of these patients had a preoperative transthoracic echocardiography suitable for speckle-tracking analysis and were included in this analysis (first patient in November 2007). The composite endpoint included all-cause death, LV assist device (LVAD) implantation, and heart transplantation. Mortality data were retrieved from the National Death Index; follow-up data from patients after heart transplantation and LVAD implantation were obtained from medical records and through telephone inquiries. No patients were lost to follow-up. The outcome analysis was conducted considering preoperative clinical and echocardiographic parameters, including STE parameters (see Figure 1 for the study flowchart). This study was performed according to the principles of the Declaration of Helsinki and was approved by the local ethics committee (EA2/177/20, 12/14/2020).

### Surgical Technique

All the SVR procedures were performed using a median sternotomy approach under cardiopulmonary bypass. CABG surgery (*n* = 113, 71.5%), mitral valve repair or replacement (*n* = 37, 23.4%), or aortic valve replacement (*n* = 9, 5.7%) was performed during the index procedure. The majority of patients underwent a modified Dor procedure without a patch (16, 17) with one or more Fontan sutures along the aneurysm perimeter. In 17 patients (10.8%), a Dor procedure (2) using endoventricular circular patch plasty was performed.

### Transthoracic Echocardiography

All the patients underwent preoperative transthoracic echocardiography using Vivid-7 and Vivid-E9 ultrasound machines (GE Vingmed Ultrasound, Horton, Norway); the data were stored in the institutional data repository. All the images were analyzed retrospectively by a single investigator (ON) for the purpose of this study. LV end-diastolic and end-systolic volumes were measured in the apical 2- and 4-chamber views; EF was obtained using the modified biplane Simpson method (18). The assessment of the diastolic function of the LV was performed for 138 patients using parameters of transmitral flow by pulse-wave Doppler and, where available, using additional parameters according to current guidelines (19). The LV shape was assessed in the apical 2-chamber view based on the classification proposed by Di Donato (10): aneurysmal (type 1), with a clear border between the aneurysm and the residual myocardium in both LV walls; intermediate (type 2), with a clear border on only one wall; and globally akinetic (type 3). The LV sphericity index was calculated as the LV end-diastolic short axis/long axis dimension ratio in the apical 4-chamber view. Segmental wall motion was assessed visually and expressed as a score ranging from 1 to 4 (normal to dyskinetic) (18), with the global wall motion score index (WMSI) and basal WMSI as the average score across all the evaluated segments. Predischarge postoperative 2D echocardiography was available for 71 patients with no STE data acquired; only studies with an assessment of LV volumes and EF were considered.

### Speckle-Tracking Echocardiography

Speckle-tracking analysis was performed using the EchoPac software version 201 (GE Vingmed Ultrasound A/S, Norway). Longitudinal parameters of LV function were obtained for all the 158 patients from apical 2-, 3-, and 4-chamber views by manual tracking of the endocardial border at an average frame rate of 60 ± 11 fps. The longitudinal parameters were measured according to the current recommendations using the 18-segment LV segmentation model (20). Only segments with satisfactory tracking throughout the entire cardiac cycle were included in the analysis. Echocardiographic studies with inadequate speckle tracking in ≥ 2 neighboring segments within one apical view, with an overall poor image quality, or with <3 apical views per study were not included in this analysis. The mean number of segments analyzed for GLS was 17 ± 2 of 18 possible segments. In 25 studies (15.8%), one or more apical segments were not suitable for analysis. The parameters assessed were peak systolic strain, end-systolic strain, peak strain during the entire cardiac cycle, and peak systolic strain rate. End-systole was defined visually by aortic valve closure in the apical long-axis view or by pulsed-wave Doppler of the aortic valve flow. GLS was calculated as the average strain of all visible segments. Basal longitudinal strain (BLS) was calculated as the average strain of six basal segments. Postsystolic shortening (PSS) of segments was considered if ≥ 20% of shortening occurred at >90 ms after aortic valve closure (21). Mechanical dispersion was calculated for the left ventricle as a whole and for the basal level of the left ventricle as one SD of the mean time-to-peak longitudinal strain of the assessed segments (22).

### Short-Term Follow-Up

The short-term clinical and echocardiographic follow-up was available for 43 patients at a median of 10 months [interquartile range (IQR): 6–12.7 months] after SVR. At this time, data on the NYHA class were collected and 2D echocardiography with a quantification of LV volumes and EF was performed. The wall motion of basal LV segments with initial hypo- or akinesia was evaluated at the follow-up. The function of midventricular and apical segments was not compared, as the LV geometry changes after SVR and segments at the follow-up might not represent the same segments as at baseline. At the follow-up, STE with quantification of LV longitudinal parameters was performed in 27 patients.

### Statistical Analysis

All the variables are presented as mean (±SD) or median (with IQR), where applicable. Categorical variables are presented as numbers with percentages. Differences between continuous variables were assessed with the paired *t*-test or the Wilcoxon sign-rank test as appropriate, differences between qualitative variables with the chi-squared test, or the McNemar test as appropriate. The univariate and multivariate Cox proportional hazards regression analyses were applied to assess risk factors for the combined outcome in the total population. Due to the low number of events and the strong correlation between the echocardiographic parameters, it was not possible to run a comprehensive Cox regression model including several echocardiographic parameters. We evaluated individual echocardiographic parameters separately, adjusted for important clinical variables, by adding the respective echocardiographic variable to the baseline clinical model. Variables included in the model were selected on the basis of their clinical importance and their possibility to confound parameters of interest. The receiver operating characteristic (ROC) analysis with Youden's index was applied to determine the optimal cutoff values for predicting 5-year outcomes in the population that completed the 5-year follow-up. Furthermore, the Kaplan–Meier analysis was performed for the whole population to estimate the differences in event-free survival between the groups based on the obtained cutoff values, with a maximum observation period of 10 years. Interobserver variability of STE parameters was assessed for 20 randomly selected studies evaluated by two independent investigators (ON and YH) and expressed as interclass correlation coefficient (ICC) and coefficient of variation (CV). Statistical significance was defined as *p* < 0.05. The data were analyzed using SPSS, version 25 (SPSS Incorporation, Chicago, Illinois, USA), and the Kaplan–Meier curves were created using Rstudio version 1.1.463 (Rstudio, PBC, Boston, Massachusetts, USA).

## Results

### Patient Characteristics and Preoperative Echocardiographic Data

The baseline patient characteristics and the univariate analysis of their association with the combined outcome are shown in Table 1. Around two-thirds of the patients had an LV shape that did not correspond to the classical aneurysmal shape, with 54.4% having an intermediate LV shape and 13.3% having a globally akinetic LV shape. The following general STE patterns were identified: (1) mean basal strains were greater (more negative) than mean global strains (*p* < 0.0001 for all the types of strains); (2) mean peak strains throughout the cardiac cycle were greater (more negative) than mean systolic and end-systolic strains, with the difference being more pronounced for global strains than for basal strains (*p* < 0.0001 for all the types of strains); (3) around 40% of all the LV segments and around 20% of the basal LV segments (*p* < 0.0001) had a PSS pattern with peak strain registered after aortic valve closure; (4) the mechanical dispersion of the basal LV segments was slightly lower than the mechanical dispersion of the entire left ventricle (*p* < 0.0001). These patterns were more prominent in patients with an aneurysmal LV shape and less prominent in patients with a globally akinetic LV shape (Supplementary Table 1).

**Table 1:** Patient baseline characteristics and the univariate analysis for predicting the combined outcome.

**Parameter**	**All patients**	**Univariate HR for combined outcome**		
	***N =* 158**	**HR**	**95% CI**	***P*-value**
Age, years (HR for 10 years)	62 ± 11.5	1.3	1.1–1.6	0.015
Female	41 (26%)	1.19	0.7–2.0	0.5
BMI, kg/m^2^	28 ± 5	1.0	0.96–1.1	0.78
Diabetes mellitus	45 (28.5%)	1.7	1.03–2.8	0.038
Arterial hypertension	108 (68.4%)	1.58	9.9–2.7	0.1
Atrial fibrillation	18 (11.4%)	3.35	1.8–6.4	0.0002
Chronic kidney disease	30 (19%)	2.25	1.3–3.8	0.003
Plasma creatinine, mg/dL (HR for 0.1 mg/dL)	1.04[0.88; 1.3]	2.3	1.3–3.8	0.003
New York Hear Association (NYHA) functional class III–IV	141 (89.2%)	1.72	0.5–5.5	0.36
Time since MI, years	2.7 [0.3; 12.9]	1.06	1.02–1.1	0.001
Previous heart surgery	11 (7%)	2.03	0.9–4.5	0.08
Intraoperative variables				
Endoventricular patch	17 (10.8%)	0.57	0.2–1.4	0.23
Thrombectomy	32 (20.3%)	0.94	0.5–1.9	0.86
CABG	113 (71.5%)	0.88	0.5–1.5	0.63
Valve surgery	44 (27.8%)	1.72	1.1–2.8	0.03
MV repair/replacement	37 (23.4%)	1.45	0.9–2.4	0.16
AV replacement	9 (5.7%)	2.15	0.98–4.7	0.06
Cross-clamp time, min	78 ± 36	1.002	1.0–1.01	0.53
Perfusion time, min	136 ± 80	1.12	1.005–1.01	0.004
Echocardiography				
LV shape type: 1 (aneurysmal) 2 (intermediate) 3 (globally akinetic)	51 (32.2%) 86 (54.4%) 21 (13.3%)	– 1.68 3.2	– 0.93–3.0 1.58–6.5	– 0.086 0.001
LV EDDI, cm/m^2^ (HR for 10 cm/m^2^)	3.1 [2.8; 3.4]	1.64	1.1–2.5	0.027
LV ESDI, cm/m^2^ (HR for 10 cm/m^2^)	2.4 [1.9; 2.7]	2.02	1.4–3.0	0.001
LV EDVI, mL/m^2^ (HR for 10 mL/m^2^)	104 [83; 134]	1.05	0.99–1.1	0.09
LV ESVI, mL/m^2^ (HR for 10 mL/m^2^)	68 [52; 96]	1.06	0.99–1.13	0.09
SI (HR for 0.1)	0.61[0.55; 0.67]	1.66	1.2–2.6	0.001
LV EF,% (HR for 5%)	34 ± 9	0.89	0.77–1.01	0.07
LV FS,% (HR for 5%)	23 [15; 29]	0.76	0.66–0.88	0.0002
CI (Doppler), L/min/m^2^	1.84[1.48; 2.17]	0.83	0.5–1.4	0.47
WMSI	1.9 [1.7; 2.2]	1.6	0.79–3.1	0.2
Basal WMSI	1.33[1.0; 1.5]	2.2	1.1–4.5	0.035
LV mass index, g/m^2^	137[114; 163]	1.005	1.0–1.01	0.09
RVOTI, mm/m^2^	14.9[13.3; 16.4]	1.03	0.95–1.1	0.5
TAPSE, mm	17.3 ± 4	0.97	0.9–1.03	0.28
RV FAS, %	38.7 ± 10	0.99	0.97–1.02	0.53
Diastolic dysfunction grade^*^				
1 2 3	75 (57.3%)21 (16%)35 (26.7%)	– 1.5 1.8	– 0.7–3.1 0.99–3.2	– 0.26 0.056
LAVI, mL/m^2^ (HR for 10 mL/m^2^)	41 [34; 55]	1.2	1.03–1.4	0.02
MR 2+	45 (28.5%)	1.62	0.99–2.7	0.055
GLSp, %	−7.6[−9.7; −5.8]	1.1	1.002–1.2	0.045
GLSs, %	−5.4 [−7.7; −3.6]	1.05	0.97–1.1	0.21
GLSes, %	−5.2[−7.5; −3.3]	1.07	0.98–1.16	0.12
GLSR, s^−1^	−0.53 [−0.64; −0.45]	3.4	0.72–16.5	0.12
BLSp, %	−11 ± 3.2	1.09	1.01–1.17	0.03
BLSs, %	−9.9 ± 3.4	1.06	0.99–1.1	0.07
BLSes, %	−9.3 ± 3.4	1.09	1.01–1.16	0.02
BLSR, s^−1^	−0.7[−0.8; −0.59]	3.7	0.88–15.4	0.08
Proportion of LV segments with PSS pattern, %	41 [24; 53]	1.0	0.98–1.0	0.29
Proportion of LV basal segments with PSS pattern, %	17 [17; 50]	1.0	0.99–1.01	0.9
MD, ms	76 [58; 107]	1.005	1.0–1.01	0.1
Basal MD, ms	69 [55; 81]	1.01	1.0–1.02	0.003

*AV, aortic valve; BLSp, peak basal longitudinal strain; BLSs, systolic basal longitudinal strain; BLSes, end-systolic basal longitudinal strain; BLSR, basal systolic longitudinal strain rate; BMI, body mass index; CABG, coronary artery bypass graft; CI, cardiac index; GLSp, peak global longitudinal strain; GLSs, systolic global longitudinal strain; GLSes, end-systolic global longitudinal strain; GLSR, global systolic longitudinal strain rate; E/A, early to late diastolic transmitral flow velocity; EDDI, end-diastolic diameter index; EDVI, end-diastolic volume index; EF, ejection fraction; ESDI, end-systolic diameter index; ESVI, end-systolic volume index; FS, fractional shortening; LAVI, left atrial volume index; LV, left ventricular; LVAD, left ventricular assist device; MD, mechanical dispersion; MI, myocardial infarction; MR, mitral regurgitation; MV, mitral valve; PSS, post-systolic shortening; RV FAS, right ventricular fractional area shortening; RVOTI, right ventricular outflow tract index; SI, sphericity index; TAPSE, tricuspid annular plane systolic excursion; WMSI, wall motion score index*.

*Data are presented as mean ± SD, median [interquartile range], n (%)*.

* *Proportion does not represent the total population (n = 131)*.

### Change in Volumetric Echocardiographic Parameters Immediately After Svr

Predischarge postoperative echocardiography was performed in 71 patients at a median of 7 days (IQR: 4–14 days) after SVR and demonstrated a reduction in LV end-diastolic and end-systolic volume indexes by 31.8 ± 18.7% and 38.7 ± 19%, respectively, compared to the preoperative values (*p* < 0.0001). EF increased from a mean of 31.9 ± 10% to 39.3 ± 9.5% (*p* < 0.0001) (Supplementary Table 2).

### Clinical and Echocardiographic Data at the Short-Term Follow-Up

The clinical characteristics of patients who had the short-term follow-up (*n* = 43) are shown in Supplementary Table 3.

The proportion of patients with the NYHA classes III and IV decreased significantly at this time (from 83.7 to 25.5% of patients, *p* = 0.002). The global two-dimensional and STE parameters improved: LV volumes reduced and EF and global WMSI improved, systolic and end-systolic GLS improved (became more negative), and the proportion of LV segments with a PSS pattern decreased (Table 2). However, no significant improvement in basal LV function, as assessed by conventional and STE, was demonstrated. Despite a significant decrease in tricuspid annular plane systolic excursion (TAPSE), there was no change in right ventricular size and fractional area shortening at follow-up.

**Table 2:** Echocardiographic parameters at the short-term follow-up after SVR.

**Parameter**	***N* pairwise**	**Baseline *N =* 43**	**At follow-up *N =* 43**	***P*-value**
Heart rate, bpm	43	72 ± 12	70 ± 10	0.35
LV EDDI, mm/m^2^	43	3.0 [2.6; 3.3]	2.9 [2.6; 3.3]	0.08
LV ESDI, mm/m^2^	43	2.5 [2.0; 2.7]	2.2[1.8; 2.7]	0.03
LV EDVI, mL/m^2^	43	103 [88; 128]	75 [67; 101]	<0.0001
LV ESVI, mL/m^2^	43	67 [56; 90]	45 [35; 63]	<0.0001
LV EF, %	43	33.1 ± 10	41.4 ± 11	<0.0001
LV FS, %	43	21.6 ± 7.6	22 ± 8.8	0.72
WMSI	43	2.0 [1.7; 2.2]	1.56[1.4; 1.8]	<0.0001
Basal WMSI	43	1.33 [1.2; 1.5]	1.23[1.0; 1.5]	0.44
CI (Doppler), L/min/m^2^	25	1.66 [1.4; 2.1]	2 [1.6; 2.4]	0.19
LAVI, mL/m^2^	38	43 [36; 57]	42 [35; 52]	0.03
SI	42	0.6 ± 0.1	0.69 ± 0.1	<0.0001
RVOTI, mm/m^2^	40	14.8 (13, 16)	14.7[12; 16]	0.33
TAPSE, mm	25	18 ± 4.6	13.7 ± 3.1	0.001
RV FAS, %	34	38.1 ± 12	38.1 ± 10	0.99
Diastolic dysfunction grade				
1 2 3	30	17 (39.5%) 4 (9.3%) 9 (20.9%)	15 (34.9%) 5 (11.6%) 10 (23.3%)	0.49
MR 2+	40	14 (35 %)	10 (25 %)	0.34
**Speckle-tracking echocardiography**				
GLSes, %	27	−5.7 ± 2.9	−7.3 ± 3.8	0.012
GLSR, s^−1^	27	−0.56 ± 0.2	−0.62 ± 0.2	0.06
BLSes, %	27	−9.6 ± 3	−9.7 ± 4	0.67
BLSR, s^−1^	27	−0.75 ± 0.2	−0.79 ± 0.2	0.47
Proportion of LV segments with PSS, %	25	41 [18; 53]	24[8; 41]	0.045
Proportion of basal segments with PSS, %	25	33 [17; 38]	24[8; 41]	0.36
MD, ms	27	81 [63; 114]	75[58; 91]	0.39
Basal MD	27	69 [56; 84]	62[53; 86]	0.09

*BLSp, peak basal longitudinal strain; BLSs, systolic basal longitudinal strain; BLSes, end-systolic basal longitudinal strain; BLSR, basal systolic longitudinal strain rate; CI, cardiac index; GLSp, peak global longitudinal strain; GLSs, systolic global longitudinal strain; GLSes, end-systolic global longitudinal strain; GLSR, global systolic longitudinal strain rate; E/A, early to late diastolic transmitral flow velocity; EDDI, end-diastolic diameter index; EDVI, end-diastolic volume index; EF, ejection fraction; ESDI, end-systolic diameter index; ESVI, end-systolic volume index; FS, fractional shortening; LAVI, left atrial volume index; LV, left ventricular; LVAD, left ventricular assist device; MD, mechanical dispersion; MR, mitral regurgitation; PSS, post-systolic shortening; RV FAS, right ventricular fractional area shortening; RVOTI, right ventricular outflow tract index; SI, sphericity index; SVI, stroke volume index; TAPSE, tricuspid annular plane systolic excursion; WMSI, wall motion score index*.

*Data are presented as mean ± SD, median (IQR) or n (%)*.

**Figure 2:**
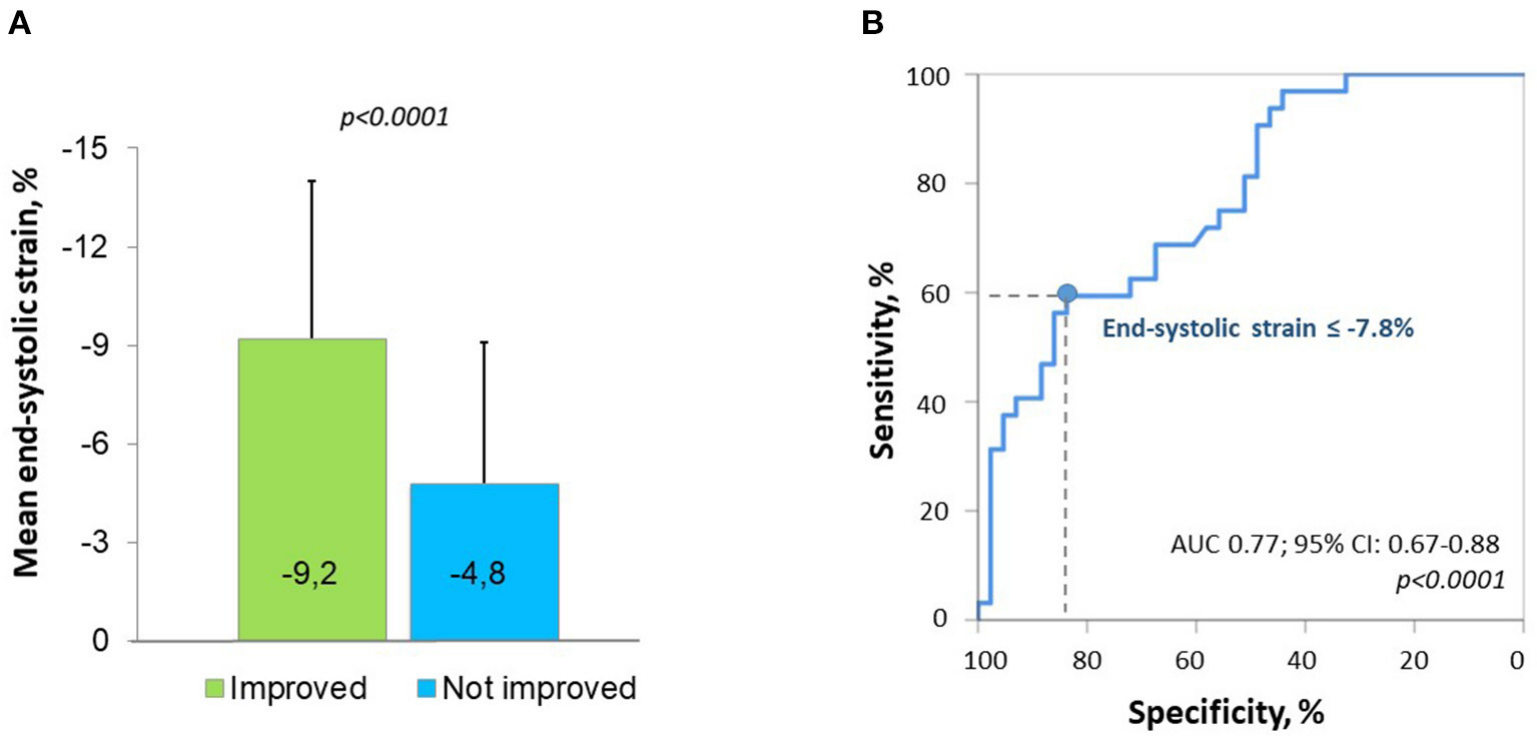
Preoperative segmental longitudinal end-systolic strain of left ventricular (LV) basal segments with initial hypo- or akinesia according to the improvement in segmental wall motion at follow-up **(A)** and the receiver operating characteristic (ROC) curve for predicting an improvement in regional wall motion by preoperative strain **(B)**.

**Figure 3:**
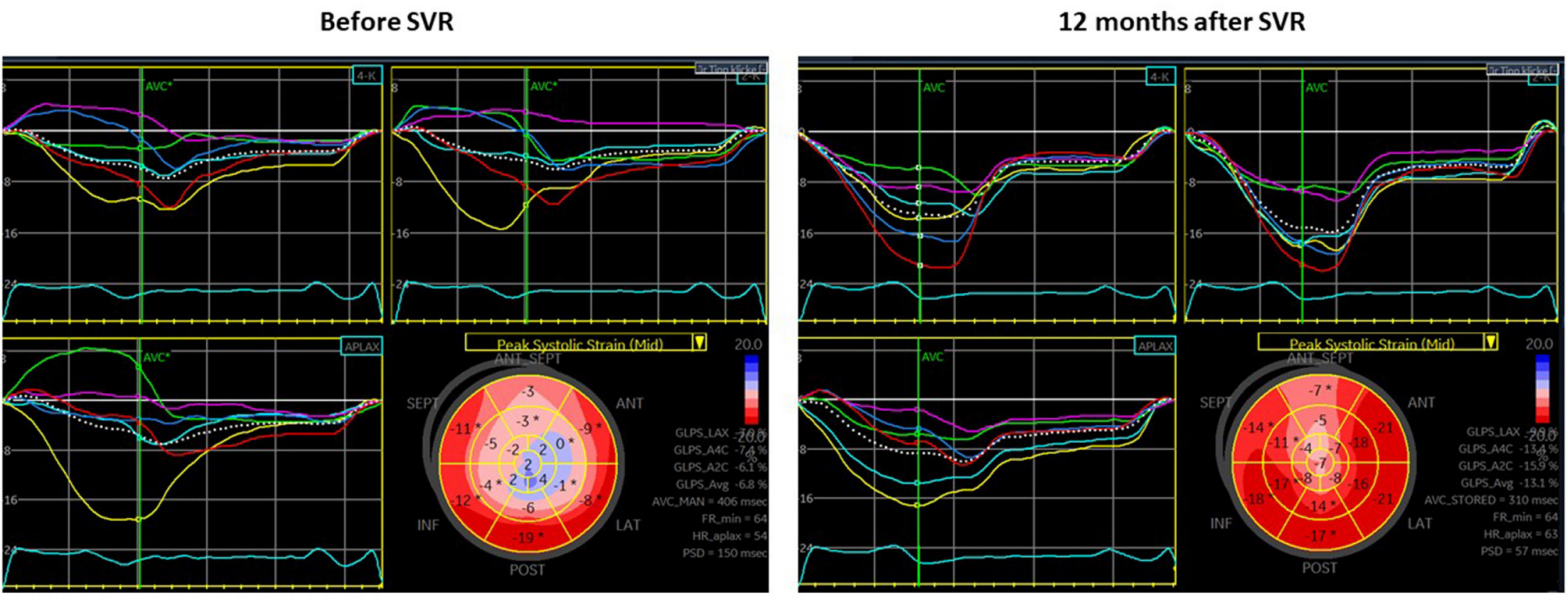
Echocardiography before surgical ventricular restoration (SVR) (left panel) and at the short-term follow-up (right panel) in a patient (65 years old, male) with end-systolic basal longitudinal strain (BLS) (-6.8%) and intermediate LV shape before SVR [without patch, no concomitant coronary artery bypass graft (CABG), or valve surgery]. At 12 months after SVR, there is a valuable improvement in EF (from 30 to 47%) and end-systolic global longitudinal strain (GLS) (from −6.8 to −13.1%), a decrease in LV end-diastolic volume (LVEDV) (from 226 to 124 ml) and LV end-systolic volume (LVESV) (from 159 to 66 ml) as well as decrease in mechanical dispersion (150 to 57 ms).

Among the basal segments with preoperative hypokinesia (*n* = 75) or akinesia (*n* = 7), an improvement in the wall motion score of ≥1 at follow-up (hypokinesia to normokinesia or akinesia to hypo- or normokinesia) was observed in 29 initially hypokinetic segments (38.7%) and in 3 initially akinetic segments (42.8%). The ROC analysis to predict the improvement in segmental wall motion at the short-term follow-up was performed for various longitudinal strain parameters including the presence of a PSS pattern (Supplementary Figure 1). End-systolic strain demonstrated the highest predictive value in the ROC analysis, with an optimal cutoff of −7.8% for predicting an improvement in segmental motion with a specificity of 83.7% and a sensitivity of 59.4% (area under the curve (AUC) 0.77, *p* < 0.0001) (Figure 2). Segments whose motion had improved at the follow-up had a less impaired (more negative) longitudinal end-systolic strain than segments that did not improve (−9.2 ± 4.8% vs. −4.8 ± 4.3%, *p* < 0.0001). No significant difference in the PSS pattern was found in segments that had improved at the follow-up compared to those that did not improve. An example of improved LV mechanics at the 12 months follow-up is shown in Figure 3.

### Outcome Data

Eight patients died in the hospital within 30 days after the surgery. During a median follow-up period of 5.1 years (IQR: 1.6–8.7 years), 67 patients died (42.4%), 9 patients (5.7%) received an LVAD, and 2 patients underwent heart transplantation. The combined outcome (LVAD implantation, heart transplantation, or death) occurred in 68 patients (48%). Overall, the event-free survival rate at 1 year was 84.1% (95% CI: 77.5–89%), at 5 years 69.4% (95% CI: 61–76.4%), and at 10 years 48.3% (95% CI: 38–57.8%). The median event-free survival time was 9.5 years (95% CI: 7.6–11.4%). Overall, the 5-year survival rate was 72.1% (95% CI: 63.8–78.8%) and the 10-year survival rate was 49.3% (95% CI: 40–58.8%). There was no difference in event-free survival between men and women, as demonstrated by the Kaplan–Meier analysis (log-rank *p* = 0.5) and the Cox regression analysis (age-adjusted HR for female sex 1.02; 95% CI: 0.6–1.7; *p* = 0.93). The era effect based on the release date of guidelines for the treatment of HF (25) also was not found to have a significant association with an outcome (age- and sex-adjusted HR 1.11; 95% CI: 0.83–1.48, *p* = *0.49*).

### Echocardiographic Predictors of the Combined Outcome

The univariate analysis suggests that several conventional and STE parameters were associated with the combined outcome (Table 1). The multivariate model of clinical variables (Supplementary Table 4) shows that time since myocardial infarction, diabetes mellitus, and atrial fibrillation were strongly associated with an outcome, whereas age, sex, and NYHA class did not demonstrate a significant association. After adjusting for clinical variables, LV volumes and dimensions as well as EF, fractional shortening (FS), sphericity index, akinetic LV shape, and grade 2 LV diastolic dysfunction were found to be associated with an outcome (Table 3). LV function assessed at the basal level, LV end-systolic diameter index, and FS demonstrated a greater improvement of the baseline model and showed a stronger association with an outcome. Global and BLS adjusted to clinical variables were associated with an outcome comparable to conventional echocardiographic parameters. End-systolic BLS demonstrated a stronger association with an outcome compared to BLS measured at other time points and also compared to GLS. Notably, global and basal WMSI were not associated with an outcome in the multivariate model.

**Table 3:** The multivariate analysis of preoperative echocardiographic parameters for predicting the combined outcome.

**Parameter**	**Baseline clinical multivariate model (χ^2^ = 36.2)** **Age, sex, NYHA ≥3, time since MI, diabetes mellitus, creatinine, atrial fibrillation**				
	**HR**	**95 % CI**	***P*-Value for HR**	**Change from the baseline model (χ^2^)**	***P*-value for change from the baseline model**
LV EDDI, 10 mm/m^2^ increase	1.8	1.1–3.1	0.025	4.8	0.03
LV ESDI, 10 mm/m^2^ increase	2.2	1.3–3.7	0.003	8.8	0.003
LV EDVI, 10 mL/m^2^ increase	1.08	1.01–1.15	0.023	4.7	0.03
LV ESVI, 10 mL/m^2^ increase	1.1	1.02–1.18	0.02	5.0	0.025
EF, 5 % increase	0.81	0.7–0.96	0.014	6.1	0.013
FS, 5 % increase	0.77	0.6–0.9	0.005	8.5	0.004
WMSI	1.88	0.7–4.8	0.17	1.7	0.19
Basal WMSI	2.2	0.83–5.8	0.11	2.4	0.12
LV shape					
Type 1 Type 2 Type 3	– 2.1 4.0	– 0.95–4.5 1.6–10.3	– 0.068 0.003	8.8	0.013
SI, 0.1 increase	1.4	1.1–1.8	0.014	5.7	0.02
TAPSE	0.99	0.93–1.1	0.84	0.04	0.84
Diastolic dysfunction grade					
1 2 3	– 2.6 1.9	– 1.1–6.2 0.9–3.7	– 0.035 0.1	5	0.08
LAVI, 10 mL/m^2^	1.11	0.9–1.35	0.31	1	0.32
MR 2+	1.3	0.7–2.5	0.34	0.9	0.35
GLSs, %	1.11	0.99–1.2	0.08	3.2	0.07
GLSes, %	1.12	1.01–1.28	0.046	4.1	0.04
GLSp, %	1.13	1.01–1.3	0.04	4.5	0.03
GLSR, s^−1^	6.1	0.95–38.9	0.056	3.9	0.049
BLSs, %	1.11	1.02–1.22	0.017	5.7	0.017
BLSes, %	1.13	1.03–1.24	0.008	7.0	0.008
BLSp, %	1.14	1.03–1.25	0.01	6.7	0.01
BLSR, s^−1^	7.2	1.35–38.7	0.021	5.8	0.016
Proportion of segments with PSS	1.0	1.0–1.02	0.83	0.05	0.83
Proportion of basal segments with PSS	1.0	1.0–1.01	0.92	0.01	0.92
MD, 10 ms increase	0.99	0.92–1.1	0.7	0.14	0.7
Basal MD, 10 ms increase	1.01	0.95–1.07	0.7	0.15	0.7

*BLSp, peak basal longitudinal strain; BLSs, systolic basal longitudinal strain; BLSes, end-systolic basal longitudinal strain; BLSR, basal systolic longitudinal strain rate; CI, cardiac index; GLSp, peak global longitudinal strain; GLSs, systolic global longitudinal strain; GLSes, end-systolic global longitudinal strain; GLSR, global systolic longitudinal strain rate; E/A, early to late diastolic transmitral flow velocity; EDDI, end-diastolic diameter index; EDVI, end-diastolic volume index; EF, ejection fraction; ESDI, end-systolic diameter index; ESVI, end-systolic volume index; FS, fractional shortening; LAVI, left atrial volume index; LV, left ventricular; LVAD, left ventricular assist device; MD, mechanical dispersion; MR, mitral regurgitation; PSS, post-systolic shortening; SI, sphericity index; SVI, stroke volume index; TAPSE, tricuspid annular plane systolic excursion; WMSI, wall motion score index*.

### Basal Longitudinal Strain and Event-Free Survival

**Figure 4:**
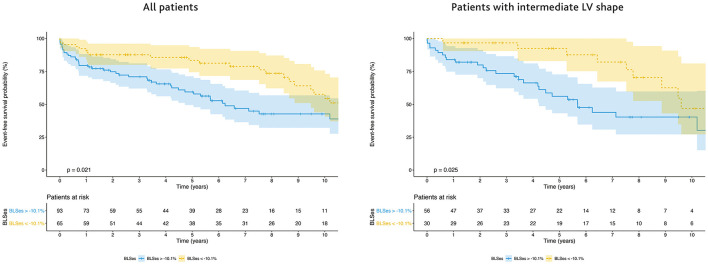
Survival free from left ventricular assist device (LVAD) and heart transplantation according to BLS in the population as a whole (left panel) and in patients with an intermediate LV shape (right panel). The Kaplan-Meier survival curves are shown as a solid line with ticks indicating censor points; shadings represent 95% CI.

There was a difference between the Kaplan–Meier event-free survival curves based on the optimal end-systolic BLS cutoff of −10.1% for predicting the 5-year outcome (sensitivity 77%, specificity 49%; AUC = 0.64, 95% CI: 0.54–0.74, *p* = 0.01), with a higher 5-year event-free survival rate in patients with BLS ≤ −10.1% (83.4%; 95% CI: 71.1–90.8%) than in those with BLS >−10.1% (59.4%; 95% CI: 55.1–47.8%) (Figure 4). The Kaplan–Meier analysis demonstrated a significant difference in the combined outcome according to the LV shape, with the longest survival in patients with an aneurysmal shape and the shortest in those with a globally akinetic shape (Supplementary Figure 2). Patients with an intermediate LV shape and end-systolic BLS ≤ −10.1% showed a higher 5-year event-free survival rate (92.4%; 95% CI: 72.8–98.1%) than those with BLS >−10.1% (56.1%; 95% CI: 40.5–69.1%) (Figure 4). No significant difference in event-free survival according to BLS was found when stratifying patients with other LV shapes.

### Interobserver Variability

Excellent interobserver agreement was demonstrated for GLS (ICC 0.96; 95% CI: 0.89–0.98; *p* < 0.0001; CV 6.6%) and BLS (ICC 0.94; 95% CI: 0.84–0.98; *p* < 0.0001; CV 6.3%). A higher interobserver variability was found for segmental LV longitudinal strain (ICC 0.93; 95% CI: 0.91–0.94; *p* < 0.0001; CV 19.6%).

## Discussion

In this study, we aimed to evaluate the association of preoperative longitudinal STE parameters with long-term outcomes in patients who underwent SVR due to an antero-apical LV aneurysm. We investigated the assumption that the regional function at the basal level of the left ventricle assessed by STE plays a role in the reverse remodeling of the left ventricle after SVR and therefore may be prognostic importance for these patients. We demonstrated that: (1) preserved preoperative segmental longitudinal strain was associated with a greater improvement in regional wall motion after SVR in segments remote from aneurysm; (2) patients with a less impaired preoperative longitudinal strain at the basal level of the left ventricle exhibited a better survival free from heart transplantation or LVAD implantation; (3) a less impaired BLS in patients with severe LV remodeling was associated with better event-free survival.

In the post-STICH era, the indication for SVR has been challenged. A preoperative evaluation of LV remodeling is performed as a part of the workup, but prognostic markers to determine a favorable outcome are lacking. In this study, we assessed the value of using two-dimensional speckle-tracking parameters to assess the LV function and found an association with outcomes in patients who underwent SVR. Although previous studies have evaluated postoperative LV remodeling using STE (26, 27), to the best of our knowledge this is the first study that assesses the predictive role of preoperative STE parameters in patients who underwent SVR. Our findings suggest that an assessment of the myocardium remote from the aneurysm with STE may provide additional prognostic information and can be useful in the preoperative evaluation of patients with antero-apical LV aneurysms. The benefit of using STE is that it does not require high expertise and has a low interobserver variability, as demonstrated by our data and previous studies (28). Since automated and semi-automated echocardiographic analyses are already being used within the scope of point-of-care ultrasonography, new diagnostic and prognostic markers derived from STE may be of additional value in various clinical scenarios. In previous studies, the assessment of LV function with STE was proven to have a higher prognostic significance compared to EF or WMSI for patients with HF (29, 30). This might also be explained by the fact that STE is not dependent on the LV shape and is not affected by geometrical assumptions.

The patients in this study had a higher mean EF (34 ± 9%) than the population that underwent SVR in the STICH study (3) (median EF 28%) and the econstructive Endoventricular Surgery returning Torsion Original Radius Elliptical (RESTORE) study (9) (mean EF 29.6%). At the same time, the median LV end-systolic volume index in our patients (68 ml/m^2^) was similar to that in patients in the RESTORE (Reconstructive Endoventricular Surgery returning Torsion Original Radius Elliptical shape to the left ventricle) study (80.4 ml/m^2^) and comparable to that in patients in the STICH trial (82 ml/m^2^). Moreover, in this study fewer patients underwent CABG (71.5%) than in the RESTORE study (95%) and the STICH trial (100%). Overall, the 5-year survival in this study (72.1%) was comparable to that of the RESTORE study (68.6%), the STICH trial, and other studies (5, 23, 31).

In line with the other reports (9, 10), this study showed an improvement in EF and a decrease in LV volumes at the short-term follow-up after SVR. A decrease in TAPSE, albeit without a change in size and function of the right ventricle, might be related to the change in the cardiac geometry and in overall heart motion due to the possible pericardial adhesion after SVR. We also observed an improvement in the NYHA functional class at the short-term follow-up. However, the preoperative NYHA functional class was not associated with an outcome in the univariate and multivariate analysis, which might be explained by the small and heterogeneous population as well as the retrospective nature of our data.

Our data allowed us to demonstrate the potential of end-systolic longitudinal strain to predict a functional improvement with a cutoff value of −7.8%. These results are consistent with the previously obtained data on the predictive role of STE in detecting viability (15). All the patients who underwent SVR in this study were fully revascularized before or during SVR. Thus, we assume that wall motion improved first due to the presence of viable myocardium in the corresponding LV segments and second due to the decrease in wall stress through LV volume reduction and LV geometry optimization. In addition, this study is the first to demonstrate that preoperative segmental longitudinal strain can be used to predict an improvement in regional LV function after SVR. In early reports of Dor et al. (32) and Di Donato et al. (33), an improvement in the regional function of LV basal inferior segments after SVR irrespective of revascularization was also observed in patients with a more prominent negative LV curvature, corresponding to a less impaired function of the myocardium remote from the aneurysm.

In their recent analysis, Castelvecchio et al. (27) evaluated LV remodeling after SVR with three-dimensional speckle-tracking in a group of 20 patients and demonstrated that GLS, BLS, and mechanical dispersion improved at the 6 months follow-up. In this study, a modest improvement in mean global peak systolic and end-systolic longitudinal strain was observed at the short-term follow-up, with some reduction in PSS but no meaningful improvement in basal LV function. This lack of improvement might be explained by the small and heterogeneous group of patients in this study: pre- and post-operative STE data were available for just 27 patients, of which only 5 patients had an aneurysmal LV shape (the most eligible patients for SVR), 19 patients had an intermediate LV shape, and 3 patients had a globally akinetic LV shape. In contrast, in the study by Castelvecchio et al. (27), all the patients were considered eligible for SVR. Although three-dimensional STE has advantages over two-dimensional STE, such as multidirectional (area) strain detection and simultaneous image acquisition for all the LV segments, two-dimensional STE has a better feasibility, higher temporal resolution, less dependent on image quality, and also less dependent on the vendor (34). In this study, we were able to use only two-dimensional data due to the retrospective nature of the analysis.

In this study, we found that parameters indicating the function of the basal LV segments, such as LV end-systolic diameter, FS, and BLS, had a stronger association with the outcome than parameters of global LV systolic function, as was demonstrated in the multivariate analysis adjusted to clinical variables. A possible explanation is that scarred segments affected by the aneurysm do not contribute much to the global LV function. On the other hand, visualization of aneurysmal apical segments in the context of LV dilatation is often complicated. Thus, speckle tracking of the apex was incomplete in around 16% of the examinations in this study. Interestingly, basal WMSI was not found to be of predictive value in the multivariate analysis, probably because STE is a more precise method for assessing myocardial function and provides a range of quantitative values compared to only four possible categories of wall motion score (normal, hypokinetic, akinetic, and dyskinetic). Although according to multivariate model BLS did not demonstrate a stronger association with an outcome compared to FS and LV end-systolic diameter, this parameter is of prognostic value and might be useful in complex preoperative assessment of patients scheduled for SVR.

As was shown in a subanalysis of the STICH trial, LV EF was not found to have predictive value for patients who underwent SVR. We believe that the function of LV regions remote from the aneurysm is a crucial factor for postoperative recovery. In this regard, involvement of basal LV segments in the scar is considered less favorable for SVR and such patients are often classified as not eligible for the procedure (5, 32, 35, 36). However, this assumption has not been confirmed in the outcome studies, along with the selection of patients based on the LV shape, which is indirectly related to the function of the basal LV myocardium. Patients with an aneurysmal LV shape corresponding to type 1 according to Di Donato (10) did not demonstrate better survival than those with other LV shapes (10, 31). The STICH trial also failed to demonstrate better outcomes in patients with only discrete anteroapical akinesia or dyskinesia (5). In contrast, this study shows a longer event-free survival in patients with an aneurysmal LV shape and a shorter survival in patients with a globally akinetic LV shape. Patients with an intermediate LV shape might be considered a grey area where additional parameters may be helpful in surgical decision-making. Our analysis showed that patients with an intermediate LV shape and less impaired BLS had a better event-free survival rate than those with more severely impaired BLS. In the STICH trial, the differentiation of left ventricles into the categories most, intermediately, and least eligible for SVR was performed on the basis of the distribution of aneurysmal segments, and not on the function of the residual myocardium. Furthermore, there was a prevalence of a kinetic segments at the basal level of the left ventricle in the group most eligible for SVR, and it is not clear how the regional function of the left ventricle basal segments differs between the groups (5).

This study shows the use of STE as a quantitative parameter of LV basal function that allows for objectively evaluating the residual LV myocardium. The assessment of LV basal myocardium is suggested by other research groups as a possible marker of LV functional improvement after SVR (27), although no prospective data have been previously reported. In a recent study (37), the authors demonstrated that the diastolic function after SVR improved in patients with a higher preoperative relative wall thickness of the basal posterior LV wall.

The use of STE parameters does not replace the conventional echocardiographic assessment of cardiac function and is not being proposed solely for the evaluation of patients scheduled for SVR. They should instead be considered in the complex assessment of patients' eligibility for the procedure and in the future might be implemented in an automated analysis of LV mechanics in a preoperative continuum.

## Limitations

This study had a nonrandomized, single-center observational design, which is a known limitation of most outcome studies on SVR representing real-world data. At the time of data collection, STE was already the standard technique in our institution, and maximum effort was made to obtain high-quality studies that were suitable for the STE analysis.

Surgical ventricular restoration was performed more than 10 years ago, which may have influenced the patient selection and the modification of treatment strategies, since HF medication and device therapy have changed significantly over the course of this study. However, the era effect did not show a significant association with an outcome. We also were not able to obtain data on medical treatments and device therapies in our patients at the time of follow-up. The short-term follow-up with an echocardiographic evaluation of LV remodeling was available only for a small number of patients, which is another potential source of bias.

All the studies and STE analyses were performed using an echocardiography system from a single vendor; therefore the cutoff values for other vendors may differ. However, a recent comparison of various vendors did not reveal significant differences in LV longitudinal strain in patients with HF (24). The cutoff for segmental strain associated with an improvement in wall motion at the short-term follow-up, as well as the cutoff for BLS associated with long-term event-free survival identified during our analysis was not validated in external populations. Further prospective investigations might be necessary to generalize these results.

## Conclusion

This study demonstrated that STE parameters were associated with outcomes in patients undergoing SVR. We showed that patients with less impaired BLS, which reflects the regional function of the myocardium remote from the aneurysm, had a better survival free from LVAD implantation and heart transplantation, also in the presence of severe LV remodeling. Less impaired preoperative segmental longitudinal strain at the basal level of the left ventricle was associated with a greater improvement in wall motion at the short-term follow-up. The implementation of quantitative analysis of LV mechanics using two-dimensional speckle-tracking may become an important part of the integrative approach in decision-making for SVR. Furthermore, the results of this study might be used for the continuous development of automated computational analyses of LV function. Future studies should prospectively investigate the impact of STE.

## Data Availability Statement

The raw data supporting the conclusions of this article will be made available by the authors, without undue reservation.

## Ethics Statement

The studies involving human participants were reviewed and approved by the Ethics Committee of the Charité – Universitätsmedizin Berlin. Written informed consent for participation was not required for this study in accordance with the national legislation and the institutional requirements.

## Author Contributions

All authors listed in the manuscript made a substantial contribution to this study reported in the manuscript. Each author was involved in the conception and design or analysis and interpretation of data, the drafting of the manuscript and its critical review for important intellectual content. All authors read the final version of the manuscript and approved its submission. The conception and design of the presented study were performed by ON, NS, MD, VF, and CK. Data collection was performed by ON, NS, MD, YH, JK, FH, and CK. The analysis and interpretation of data by all the co-authors. The statistical expertise by ON, JS, YH, and CK. The writing of the manuscript by ON, NS, and CK. The critical review and final approval of the article by all the co-authors.

## Funding

This study was supported by the DZHK (German Centre for Cardiovascular Research) and the BMBF (German Ministry of Education and Research).

## Conflict of Interest

The authors declare that the research was conducted in the absence of any commercial or financial relationships that could be construed as a potential conflict of interest.

## Publisher's Note

All claims expressed in this article are solely those of the authors and do not necessarily represent those of their affiliated organizations, or those of the publisher, the editors and the reviewers. Any product that may be evaluated in this article, or claim that may be made by its manufacturer, is not guaranteed or endorsed by the publisher.

## Supplementary Material

Supplementary Figure 1 The receiver operating characteristic (ROC) analysis for predicting an improvement in segmental wall motion at the short-term follow-up. **(Upper left panel)** The ROC curve for longitudinal strain and strain rate parameters. **(Upper right panel)** The ROC curve for postsystolic shortening (PSS) pattern.

Supplementary Figure 2 Event-free survival according to the left ventricular (LV) shape classification. The Kaplan–Meier survival curves are shown as a solid line with ticks indicating censor points; shadings represent 95% CI.
