# Supplementary figures and images for: Predictive Value of Two-Dimensional Speckle-Tracking Echocardiography in Patients Undergoing Surgical Ventricular Restoration
Source: Front Cardiovasc Med. 2022 Mar 21;9:824467. doi: 10.3389/fcvm.2022.824467 (PMC8978793; doi:10.3389/fcvm.2022.824467)

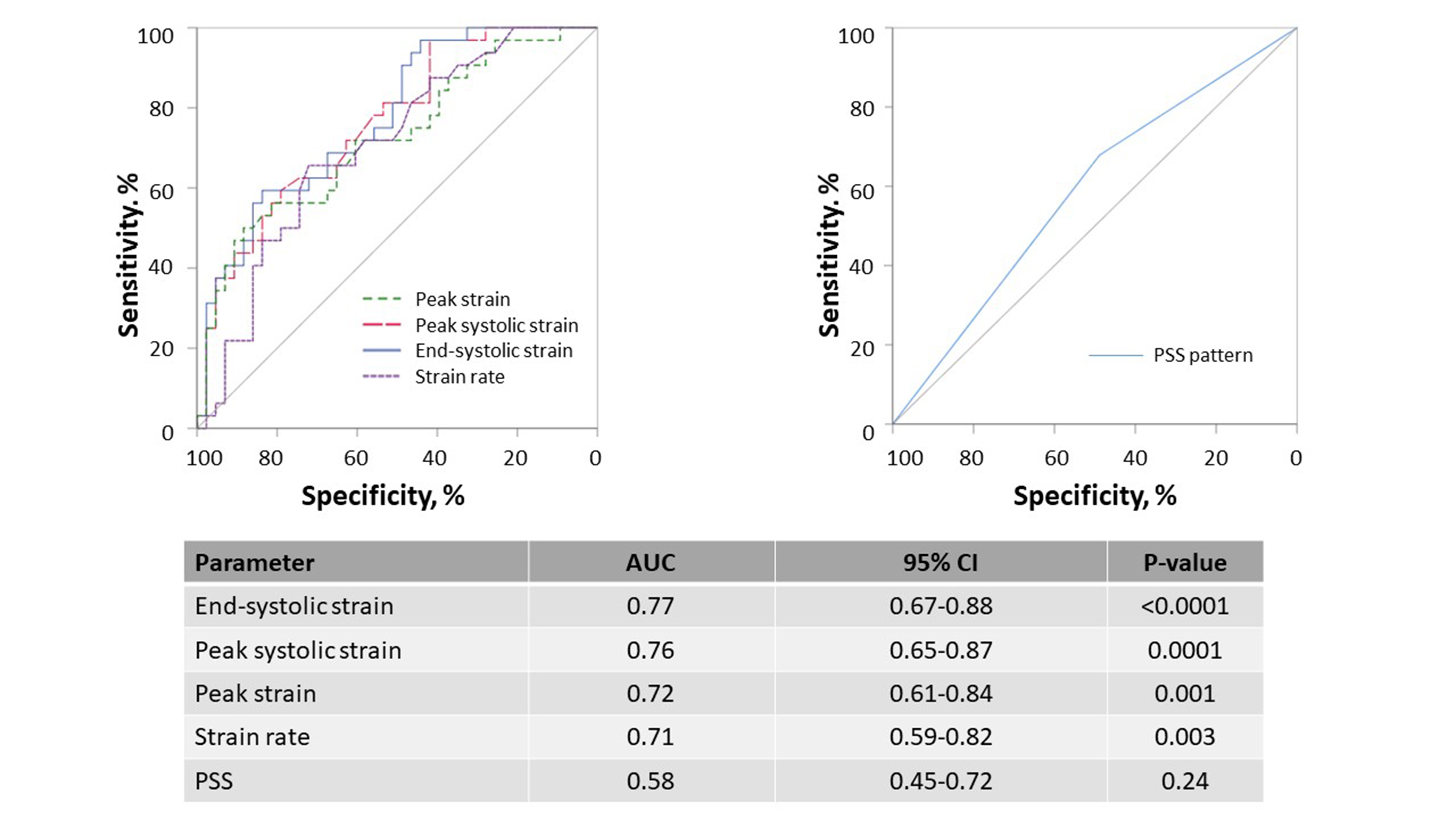

Supplement: Supplementary Figure 1 — The receiver operating characteristic (ROC) analysis for predicting an improvement in segmental wall motion at the short-term follow-up. (Upper left panel) The ROC curve for longitudinal strain and strain rate parameters. (Upper right panel) The ROC curve for postsystolic shortening (PSS) pattern. [file Image_1.JPEG]

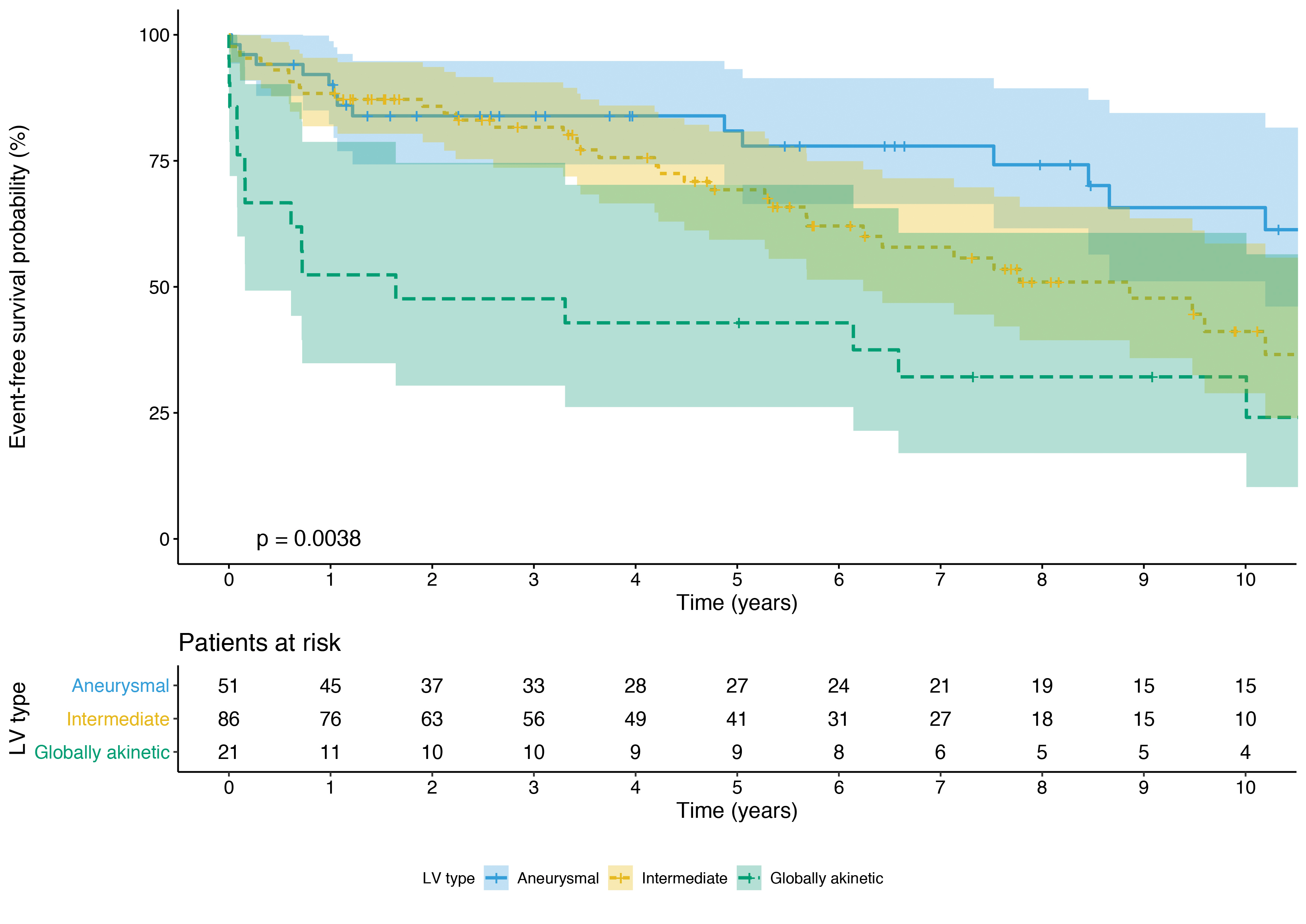

Supplement: Supplementary Figure 2 — Event-free survival according to the left ventricular (LV) shape classification. The Kaplan–Meier survival curves are shown as a solid line with ticks indicating censor points; shadings represent 95% CI. [file Image_2.JPEG]
