# Supplementary material for: Predictive Value of Two-Dimensional Speckle-Tracking Echocardiography in Patients Undergoing Surgical Ventricular Restoration
Source: Front Cardiovasc Med. 2022 Mar 21;9:824467. doi: 10.3389/fcvm.2022.824467 (PMC8978793; doi:10.3389/fcvm.2022.824467)
Supplement: Supplementary file 3 [file Data_Sheet_1.pdf]

Supplemental Table 1. Patient baseline characteristics in groups by LV shape.

Parameter	Groups by LV shape			
	Aneurysmal N=51	Intermediate N=86	Globally akinetic N=21	p-Value
Age, years	66.7±10.9	60±11.7	61.3±10.3	0.02
Women	18 (35.3)	19 (22.1)	4 (19)	0.17
BMI, kg/m^2	28.3±5.5	27.6±4.7	26.7±3.4	0.43
Diabetes mellitus	13 (25.5)	28 (32.6)	4 (19)	0.4
Arterial hypertension	38 (74.5)	58 (67.4)	12 (57.1)	0.34
Atrial fibrillation	5 (9.8)	8 (9.3)	5 (23.8)	0.16
Chronic kidney disease	9 (17.6)	18 (20.9)	21 (14.3)	0.75
Plasma creatinine, mg/dL	1.0 [0.8-1.2]	1.1 [0.9-1.3]	1.02 [0.85-1.33]	0.6
NYHA functional class III-IV	42 (82.4)	78 (86)	21 (100)	0.073
Time since MI, years	6.9 [2.4-119]	34 [3.4-166]	113 [22-214]	0.09
Previous heart surgery	2 (3.9)	4 (4.7)	5 (23.8)	0.005
**Intraoperative variables**				
Endoventricular patch	6 (11.8)	10 (11.6)	1 (4.8)	0.64
Thrombectomy	13 (25.5)	18 (20.9)	1 (4.8)	0.14
CABG	38 (74.5)	59 (68.6)	16 (76.2)	0.67
Valve surgery	8 (15.7)	24 (27.9)	12 (57.1)	0.002
MV repair/replacement	4 (7.8)	22 (25.6)	11 (52.4)	0.002
AV replacement	3 (5.9)	4 (4.7)	2 (9.5)	0.69
Cross-clamp time, min	75±34	76±33	91±48	0.19
Perfusion time, min	124±57	128±53	198±160	0.001
**Echocardiography**				
LV EDDI, cm/m^2	2.8 [2.6; 3.0]	3.1 [2.9; 3.4]	3.7 [3.4; 3.9]	<0.0001
LV ESDI, cm/m^2	1.9 [1.7; 2.1]	2.5 [2.2; 2.7]	3.0 [2.7; 3.4]	<0.0001
LV EDVI, mL/m^2	81 [76; 94]	113 [97; 134]	155 [110; 176]	<0.0001

Parameter	Groups by LV shape			
	Aneurysmal N=51	Intermediate N=86	Globally akinetic N=21	p-Value
LV ESVI, mL/m^2	48 [39; 63]	76 [63; 96]	116 [69; 133]	<0.0001
SI	0.57 [0.49; 0.62]	0.62 [0.57; 0.67]	0.76 [0.64; 0.78]	<0.0001
LV EF, %	42±8	32±7	27±8	<0.0001
LV FS, %	30 [24; 37]	21 [14; 27]	14 [12; 23]	<0.0001
CI (Doppler), L/min/m^2	1.84 [1.48; 2.14]	1.83 [1.5; 2.14]	1.85 [1.6; 2.2]	0.94
WMSI	1.69 [1.5; 1.94]	2 [1.75; 2.2]	2.25 [2.1; 2.4]	<0.0001
Basal WMSI	1.0 [1.0; 1.17]	1.33 [1.17; 1.5]	1.67 [1.5; 1.8]	<0.0001
LV mass index, g/m^2	116 [104; 147]	142 [121; 162]	170 [128; 216]	<0.0001
TAPSE, mm	18.6±3.7	16.7±4.1	16.7±4.2	0.029
Diastolic dysfunction grade*				
1	30 (72%)	41 (53.9%)	4 (28.6%)	
2	6 (14.6%)	13 (17.1%	2 (14.3%)	0.015
3	5 (12.2%)	22 (28.9%)	8 (57.1%)	
MR 2+	7 (13.7)	26 (30.2)	12 (57.1)	0.001
GLSp, %	-10 [-12.3; -7.5]	-7.2 [-8.6; -5.8]	-5.8 [-6.8; -4.7]	<0.0001
GLSs, %	-7 [-9.7; -5.1]	-5.3 [-7.2; -3.4]	-5 [-4.9; -2.5]	<0.0001
GLSes, %	-6.7 [-9.6; -4.9]	-4.7 [-6.6; -3]	-3.2 [-4.3; -2.4]	<0.0001
GLSR, s^{-1}	-0.64 [-0.78;-0.53]	-0.51 [-0.58;-0.42]	-0.46 [-0.54;-0.36]	<0.0001
BLSp, %	-13.1±2.9	-10.5±2.6	-7.9±3.0	<0.0001
BLSs, %	-12.1±3.1	-9.4±2.8	-6.6±3.2	<0.0001
BLSes, %	-11.5±3.1	-8.8±2.8	-5.9±3	<0.0001

Parameter	Groups by LV shape			
	Aneurysmal N=51	Intermediate N=86	Globally akinetic N=21	p-Value
BLSR, s^{-1}	-0.79 [-0.94; -0.66]	-0.7 [-0.77; -0.57]	-0.56 [-0.67; -0.47]	<0.0001
Proportion of LV segments with PSS pattern, %	41 [25; 53]	41 [21; 55]	47 [31; 53]	0.82
Proportion of basal LV segments with PSS pattern, %	17 [17; 33	33 [17; 50]	42 [17; 50]	0.07
MD, ms	73 [58; 100]	74 [54; 101]	85 [66; 160]	0.15
Basal MD, ms	66 [52; 74]	69 [54; 82]	83 [70; 105]	0.0002

AV – aortic valve, BLSp – peak basal longitudinal strain, BLSs – systolic basal longitudinal strain, BLSes – end-systolic basal longitudinal strain, BLSR –basal systolic longitudinal strain rate, BMI – body mass index, CABG – coronary artery bypass graft, CI – cardiac index, GLSp – peak global longitudinal strain, GLSs – systolic global longitudinal strain, GLSes – end-systolic global longitudinal strain, GLSR – global systolic longitudinal strain rate, EDDI – end-diastolic diameter index, EDVI – end-diastolic volume index, EF – ejection fraction, ESDI – end-systolic diameter index, ESVI – end-systolic volume index, FS – fractional shortening, LV – left ventricular, LVAD – left ventricular assist device, MD – mechanical dispersion, MI – myocardial infarction, MR – mitral regurgitation, MV – mitral valve, PSS – post-systolic shortening, SI – sphericity index, TAPSE – tricuspid annular plane systolic excursion, WMSI – wall motion score index.

Data are presented as the mean ± SD, median [interquartile range], or n (%).

* proportion does not represent the total population (n=41 for aneurysmal shape, n=76 for intermediate shape and n=14 for globally akinetic shape).

Supplemental Table 2. Difference between preoperative and postoperative pre-discharge echocardiographic parameters.

Parameter	N	Preoperative	Pre-discharge	P-value
LV EDDI, mm	71	31.6±5	28.5±5.1	<0.0001
LV ESDI, mm	71	24.5±5.3	22.3±5.3	<0.0001
LV FS, %	71	22.8±8.7	22.6±7.8	0.88
LV EDVI, mL/m^2	67	125±44	81±25	<0.0001
LV ESVI, mL/m^2	67	88±39	51±22	<0.0001
LV EF, %	67	31.9±10	39.3±9.5	<0.0001

EDDI – end-diastolic diameter index, EDVI – end-diastolic volume index, EF – ejection fraction, ESDI – end-systolic diameter index, ESVI – end-systolic volume index, FS – fractional shortening, LV – left ventricular.

Supplemental Table 3. Clinical characteristics of patients included in short-term follow-up.

**Parameter**	**Patients with short-term follow-up N=43**
Age, years	58.3±12
Women	8 (18.6)
Diabetes mellitus	7 (16.3)
Arterial hypertension	29 (67.4)
Atrial fibrillation	5 (11.6)
Chronic kidney disease	6 (14)
NYHA functional class III-IV	36 (86.7)
Time since MI, years	6.1±7.9
**Intraoperative variables:**	
Endoventricular patch	9 (20.9)
Thrombectomy	15 (34.9)
CABG	22 (51.2)
Valve surgery	14 (32.6)
Cross-clamp time, min	73±36
Perfusion time, min	123±56

CABG – coronary artery bypass graft, MI – myocardial infarction.

Supplemental Table 4. Multivariate model including preoperative clinical variables for predicting combined outcome.

Variables in the Equation	Cox regression analysis $\chi2$=36.2		
	**HR**	**95% CI**	***P-value***
**Age, 10 years**	1.1	0.84-1.44	0.51
**Sex, female**	1.1	0.57-2.1	0.78
**NYHA functional class III-IV**	4.2	0.56-32	0.16
**Time since myocardial infarction, years**	1.06	1.03-1.1	0.001
**Diabetes mellitus**	2.0	1.1-3.6	0.03
**Plasma creatinine, 0.1 mg/dL**	1.05	0.99-1.1	0.095
**Atrial fibrillation**	3.2	1.5-6.9	0.003
